# Quantum Dots in Transition Metal Dichalcogenides Induced by Atomic-Scale Deformations

**DOI:** 10.1021/acsphotonics.3c01470

**Published:** 2024-01-16

**Authors:** Jannis Krumland, Stefan Velja, Caterina Cocchi

**Affiliations:** † Institute of Physics, Carl von Ossietzky Universität Oldenburg, 26129 Oldenburg, Germany; ‡ Physics Department and IRIS Adlershof, Humboldt-Universität zu Berlin, 12489 Berlin, Germany

**Keywords:** quantum dots, 1L-TMDC, deformations, molybdenum diselenide

## Abstract

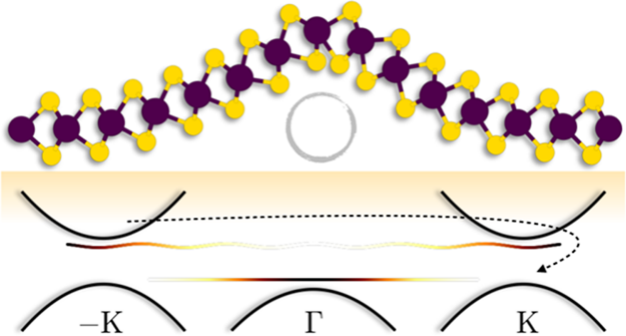

Single-photon emission from monolayer transition metal dichalcogenides requires the existence of localized, atom-like states within the extended material. Here, we predict from first-principles the existence of quantum dots around atomic-scale protrusions, which result from substrate roughness or particles trapped between layers. Using density functional theory, we find such deformations to give rise to local membrane stretching and curvature, which lead to the emergence of gap states. Having enhanced outer-surface localization, they are prone to mixing with states pertaining to chalcogen vacancies and adsorbates. If the deformation is sharp, the conduction band minimum furthermore assumes atomic and valley-mixed character, potentially enabling quantum light emission. When such structural defects are arranged in an array, the new states couple to form energetically separated sub-bands, holding promise for intriguing superlattice dynamics. All of the observed features are shown to be closely linked to elastic, deformation-induced intra- and intervalley scattering processes.

## Introduction

Engineered strain fields and point defects in monolayer transition metal dichalcogenides (1L-TMDCs) have been intensely studied in recent years for their potential applications in quantum information technology. After the first reports of single photon emission in tungsten diselenide,^1−5^ many approaches for controlled inclusion of such quantum light sources have been proposed, quickly extending to other 1L-TMDCs as well.^6−9^ The emission has often been ascribed to quantum wells induced by macroscopic, locally varying strain fields, which also function as funnels for excitons due to local band gap narrowing.^10,11^

Common strategies for generating such strain fields involve prepatterned substrates^12−15^ and gas injection between layers.^16,17^ However, the hypothesis of single-photon emission from purely strain-related quantum wells has been challenged, noting that they are too large to give rise to discrete energy levels.^18,19^ Consequently, it is possible that strain gradients only play an auxiliary role, guiding valley excitons toward trap states that are the actual sources of quantum light.^20,21^ However, the microscopic nature of these sources is not clear yet. They are possibly related to point defects such as vacancies^8,22^ or to sharp structural deformations.^18,19^ Atomic-scale deformations can be introduced by bringing the layer in contact with nanostructures or irregular substrates.^23−27^ This also includes surface roughness and trapped particles, making these defects relevant in all kinds of realistic samples. The interpretation of the corresponding experimental results often focuses exclusively on strain. However, this type of modeling is likely to be too reductive on this length scale. Other parameters have to be considered, e.g., the curvature,^28^ which can become very large in atomic-scale deflections and which breaks the symmetry of the two sandwiching chalcogen layers.

In this work, we investigate the electronic structure of atomic-scale deformations in monolayer molybdenum disulfide (1L-MoS_2_) using fully quantum-mechanical and atomistic ab initio methods. Our study encompasses both atomically sharp and relatively smooth structures, which model different experimental scenarios (adsorption on a rough substrate vs bubbling due to gas accumulation), enabling a comparative analysis of the effects of concentrated and extended regions of strain, curvature, and corresponding gradients. Adopting band unfolding techniques, we elucidate the impact of geometrical defects on the electronic structure of the material. Across both smooth and sharp structures, we anticipate the emergence of gap states and satellite states. With a lowered conduction band minimum and a reduced fundamental gap, these structural defects act as traps for both electrons and excitons. A distinguishing feature of the sharp deformation is the real-space localization of the lowest conduction states, which assume increased single-atomic  character and exhibit broken valley symmetry. These features have severe implications for the optoelectronic properties, rendering these defects possible sources of quantum light and thus unveiling the microscopic origin of single-photon emission from 1L-TDMCs. Our analysis furthermore predicts a highly position-dependent coupling between the states originating from the deformation and sulfur vacancies, inducing significant shifts in quantum dot energy levels spanning several hundred meV. Finally, we extend our study to related materials including bilayer MoS_2_ and monolayer molybdenum diselenide. Notably, our results for the latter closely mirror those obtained for 1L-MoS_2_, suggesting that our findings are largely transferable to 1L-TMDCs in general.

## Methods

The electronic structure calculations of this work are based on the density functional theory,^29^ solving the Kohn–Sham (KS) equation^30^
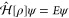
1where *E* and ψ stand for KS energies and orbitals, respectively.  is the spatially represented KS Hamiltonian, which is functionally dependent on the electron number density
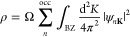
2Here, ψ_*n***K**_ is the solution of eq 1 with eigenvalue *E*_*n***K**_ associated with the band index *n* and the two-dimensional Bloch vector **K**; Ω is the base plane area of the cell, and the integral extends over the first Brillouin zone (BZ). The nonlinear problem given by eqs 1 and 2 is solved iteratively until self-consistency is reached.

The deformed geometries are constructed on the basis of supercells, which complicate the electronic structure by folding the energy bands. To recover the more intuitive and well-known description within the primitive BZ, we employ an unfolding technique.^31^ We start with the plane-wave representation *c*_*n***K**_ of a spinor-valued orbital ψ_*n***K**_
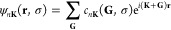
3where σ is the spin index and **G** runs over the reciprocal lattice of the supercell. The value of the spectral function  of this state for a primitive-cell vector **k** = **K** + **G**_**k**_, differing from **K** by a reciprocal superlattice vector **G**_**k**_, is then computed as
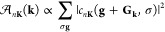
4with the sum running over the primitive-cell reciprocal lattice vectors **g**. A spin-resolved version of the spectral function
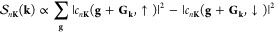
5is adopted to capture the phenomena with a corresponding sensitivity, e.g., spin-flip scattering. The state-specific  of eq 4 are joined to construct the total spectral function  of the system
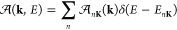
6where for a given **k**, the wavevector **K** is chosen such that **k** = **K** + **G**_**k**_ for a reciprocal superlattice vector **G**_**k**_. In practice,  is visualized by introducing an energy broadening, replacing the δ function with a narrow Gaussian.

We determine the contribution *P*_*n***K**_(**k**) of the supercell state ψ_*n***K**_ from components around the primitive-cell wavevector **k** as
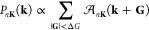
7where Δ*G* is a fixed radius (see Supporting Information, Figure S1). The valley polarization is then calculated as
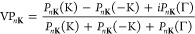
8

Usually, this quantity is defined only with respect to the K and −K valleys.^32^ However, for the specific case of 1L-MoS_2_, the Γ valence valley can also be expected to participate in hole dynamics as it is energetically close to the valence band maximum (VBM) at K. Thus, the Γ population is included in eq 8 as an imaginary component. As in the real-valued ±K-only expression, a VP_*n***K**_ close to ±1 indicates contributions from ±K, while a VP_*n***K**_ approaching 0 implies a K/–K-mixed state. When VP_*n***K**_ is close to *i*, the corresponding state is mostly **k**-localized at Γ.

## Computational Details

All calculations are performed with version 6.8 of the Quantum ESPRESSO suite.^33^ The exchange–correlation potential is approximated with the semilocal Perdew–Burke–Ernzerhof (PBE) functional,^34^ having ensured that higher-level theory in the form of hybrid functional delivers qualitatively equal results (Section S1). We employ fully (scalar-)relativistic norm-conserving SG15 pseudopotentials^35,36^ with a wave function cutoff of 40 Ry (60 Ry) for band structure calculations (geometry optimizations). Spin–orbit coupling is taken into account for the band structures. The Tkatchenko–Scheffler dispersion correction is included in the structural relaxations,^37^ which are considered converged once all components of all forces are below 10^–3^ Ha/bohr. Band structure unfolding is conducted with an in-house developed code.^47^ Computational complexity is reduced by choosing path segments parallel with the supercell lattice vectors [Figure S2a]. This choice minimizes the amount of inequivalent supercell **K** vectors onto which the **k** vectors along the path map. A symmetric scissor operator is applied to open the bandgap by a quantity given by the difference between the PBE band gap in the flat material and 2.15 eV, corresponding to the value measured when the material is adsorbed on a strongly screening substrate.^38^ For real-space visualizations of geometries, we employ XCrysDen.^39^

## Results and Discussion

### Deflection Geometries

**Figure 1:**
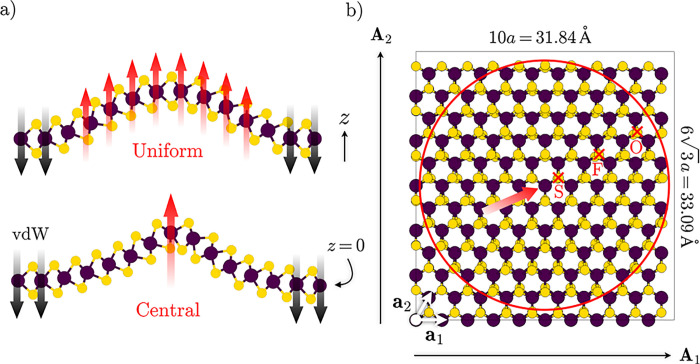
(a) Schematic side view of uniform- and central-load deformations in 1L-MoS_2_. Mo and S atoms are depicted as purple and yellow spheres, respectively. The red arrows represent the bubble-forming forces, and the black ones the van der Waals forces keeping the material pinned to the substrate on the periphery. (b) Top view of the  simulation cell spanned by the lattice vectors **A**_1_ and **A**_2_. The primitive lattice vectors of flat 1L-MoS_2_ are shown as **a**_1_ and **a**_2_. The red arrow points to the central Mo atom. All Mo atoms outside the circle are fixed at base height *z* = 0. The three crossed-out S atoms are individually removed in the study of the single S vacancies. The sites are termed “(S)ummit”, “(F)lank”, and “(O)utskirts”, as labeled.

We consider two types of atomic-scale deformations in 1L-TMDCs, termed “uniform load” (UL) and “central load” (CL) structures (Figure 1a), which result from different sorts of upward-acting forces. With UL, the force is distributed over an area, while for CL, it has a point-like character. UL models situations in which small gas accumulations exert pressure on the membrane, whereas CL corresponds to singular trapped atoms or substrate adatoms. Notably, UL results in a smooth deformation, while CL creates a sharp cusp at the center. The structures are obtained by constrained relaxation, fixing atoms at base height *z* = 0 outside a circle with a radius of 15 Å around a designated central Mo atom [Figure 1b]. In the UL case, an upward external force *F***e**_*z*_ is applied to all lower-plane S atoms inside the circle, where **e**_*z*_ is the unit vector in the *z* direction; for CL, the center Mo atom is fixed at a height *z* = *h* above the base plane. Subject to these constraints, the structures are relaxed by total force minimization. The parameters are adjusted to yield structures with an aspect ratio (height)/(radius) of about 0.16, i.e., the experimentally observed, size-independent value for nanobubbles of 1L-MoS_2_,^40^ which is the material we mainly focus on. While the resulting structures are characterized by an already large amount of strain and deformation, we obtain stable structures and bonds even with significantly stronger forces, demonstrating the robustness of the material (Figure S3). Periodic boundary conditions are applied, implying that we simulate periodic superlattices of defects. Properties of isolated entities are extrapolated along the way.

### Strain and Semiclassical Electronic Structure

**Figure 2:**
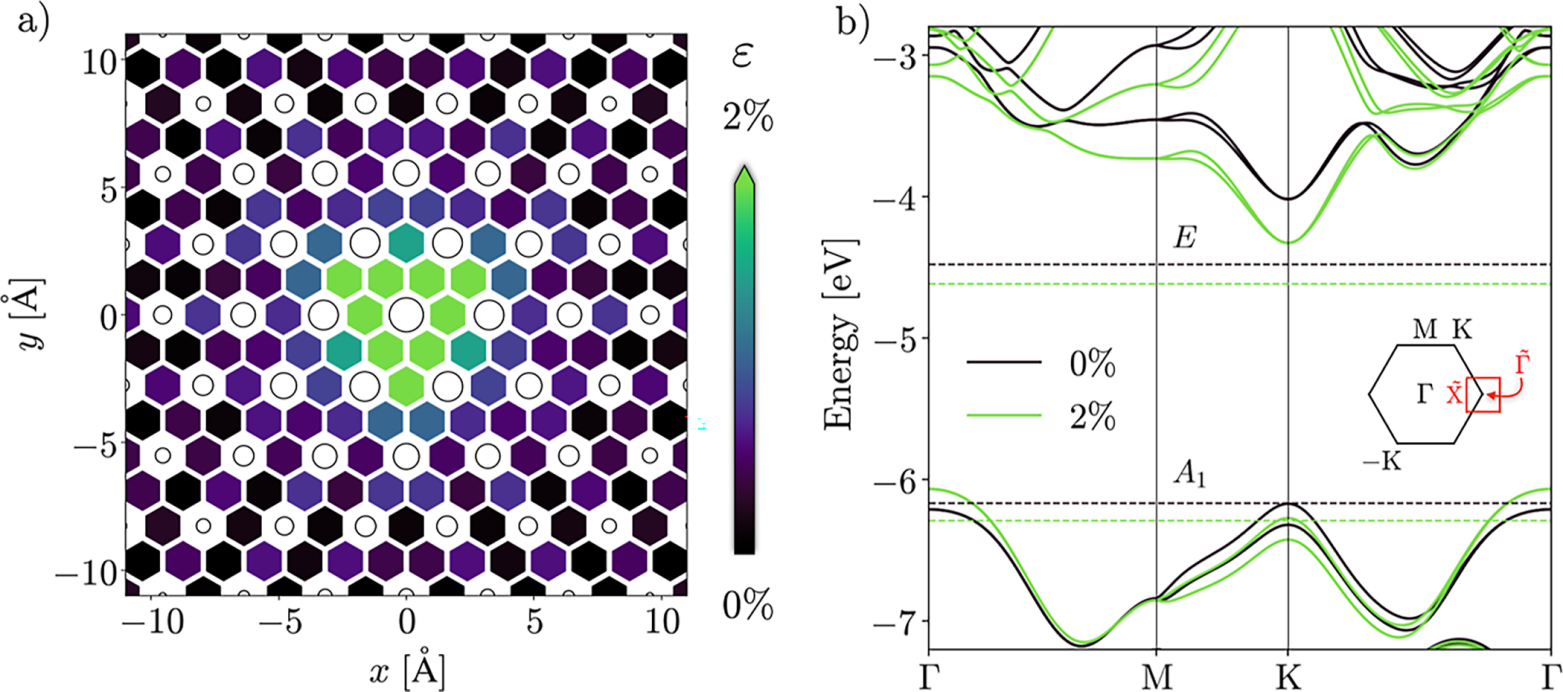
(a) Strain (ε) around the central Mo atom (*x* = *y* = 0) of the centrally loaded membrane, calculated for each pair of Mo atoms as (*d* – *d*_0_)/*d*, where *d* is their actual distance and *d*_0_ the equilibrium value. The color bar is clipped at the upper end; the strain values connecting the central Mo atom with its 6 nearest neighbors amount to 3.75%. The circles mark the positions of the Mo atoms in the *xy* plane, while their size is indicative of the *z* coordinate. (b) Band structure of 1L-MoS_2_ in the relaxed (black) and biaxially stretched (green) phases, pertaining to the outskirts and the apex, respectively. The dashed horizontal lines represent occupied (*A*_1_) and unoccupied (*E*) localized states introduced by single sulfur vacancies. The inset shows the first unit-cell BZ with the relevant high-symmetry points highlighted. The red zone pertains to the quadratic superlattice of protrusions; it is oversized for clearance.

To set the stage for the analysis of the fully atomistic electronic structures of these deformations, we first assume a conventional semiclassical viewpoint,^41^ which can be taken to describe the optoelectronic structure of larger-scale deflections. The geometry of and strain within the membrane are modeled within a continuum model, yielding a macroscopic strain field. The local strain values, in turn, determine the local band edges, giving rise to a spatially varying band gap. Here, we map out the strain in the structures by determining the Mo–Mo distances [Figure 2a]. The forces cause membrane stretching, i.e., tensile strain, with the maximum value assumed around the central Mo atom. It peaks at 3.75% for CL, quickly decaying toward the outskirts of the structure; with UL, the values are considerably lower (≤1.5%) but fall off more gently [Figure S4a]. The circumferential component of the strain decreases more rapidly than that of the radial one, giving rise to a transition from biaxial to uniaxial strain from the center of the bubble to the edge [Figure S4b]. A corresponding behavior has been observed in larger bubbles.^17^

Invoking the above-mentioned semiclassical model, we now qualitatively identify the local band structures at the summit and the peripheral regions with those of homogeneously stretched and relaxed 1L-MoS_2_, respectively [Figure 2b]. 2% of biaxial tensile strain is applied, which is intermediate with respect to the highest values assumed in the UL and CL structures. The main effects of tensile strain are the lowering of both the conduction band minimum (CBm) and the VBM at K, to the point where the latter is overtaken by the rising Γ valley. Thus, the band gap turns indirect near the apex, which manifests in local photoluminescence quenching.^11,42,43^ The CBm is lowered, and the electronic gap is substantially reduced, which also translates into the optical gap.^44^ Hence, the deformation constitutes a robust trap for electrons and excitons.

### Quantum-Mechanical Electronic Structure

#### Intravalley Scattering

**Figure 3:**
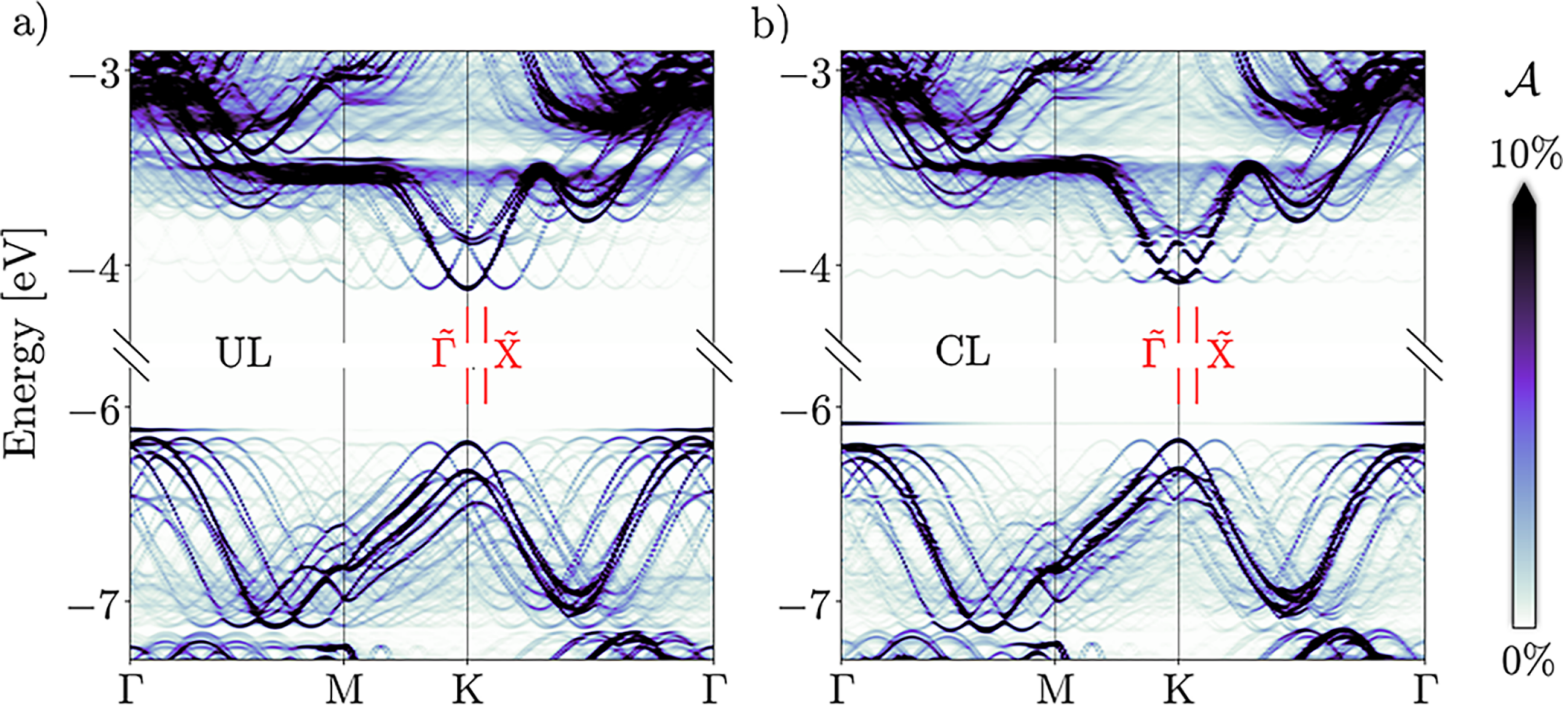
Unfolded band structure for (a) UL and (b) CL deformations.  is the spectral weight, and  and  are high-symmetry points of the mini BZ associated with the deflection superlattice [cf. Figure 2b].

Contrasting this macroscopic picture, we now analyze the results of fully atomistic simulations. Unfolded band structures of the entire cell, including both the protrusion and the flat periphery, exhibit a number of qualitative differences with respect to those of the flat systems (Figure 3). While the inhomogeneous tensile strain also decreases the direct gap at K by 90 meV (UL) or 70 meV (CL), this reduction is significantly smaller than that in the flat system (220 meV). Consequently, trap levels from atomic-size deformations are shallower than those from mesoscopic strain-induced potential wells. Like in the larger structures, the VBM wanders toward Γ but has a markedly different appearance. A flat gap state is split off and energetically separated from the strongly perturbed continuum of states underneath. The influence exerted on the K valleys is not as dramatic but still significant, especially with CL. Each valley is surrounded by replica bands of varying strengths. In the following, we discuss each of these features in detail.

**Figure 4:**
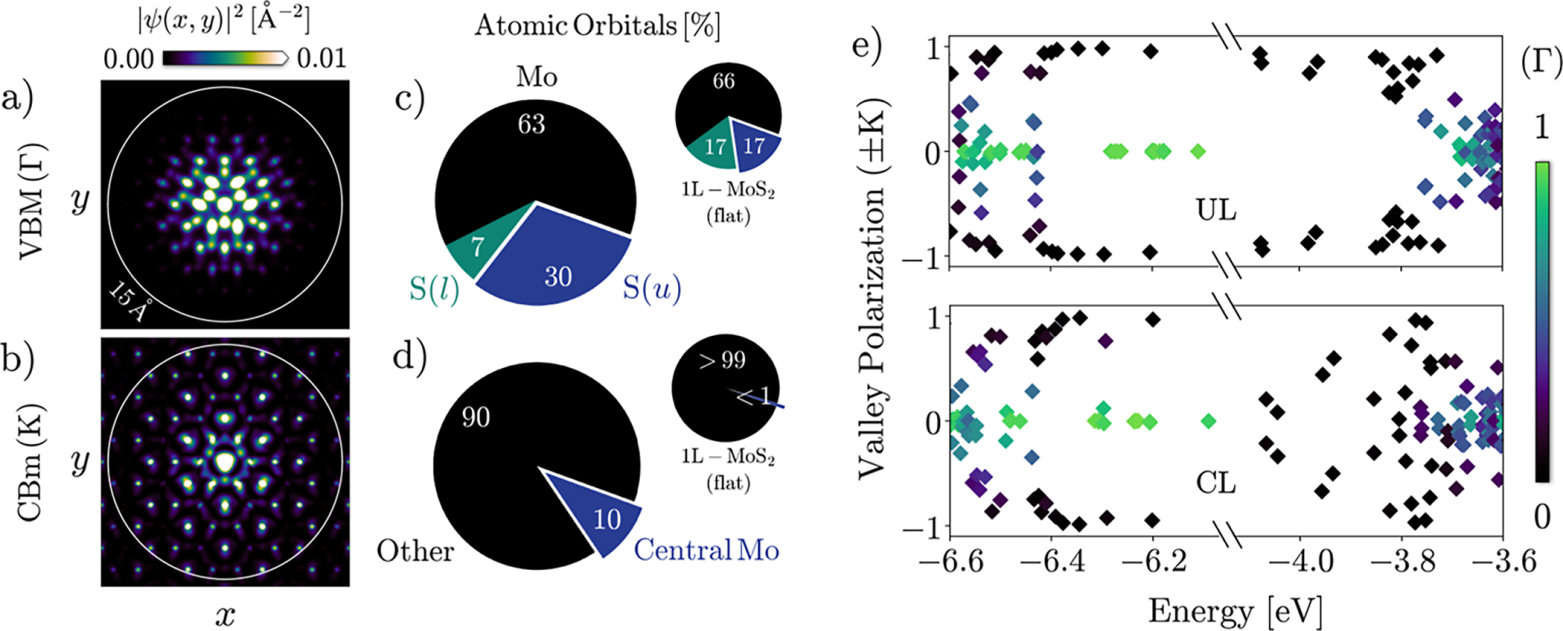
Wave function analysis. (a,b) Real-space representations of the VBM and the CBm in the CL structure. The *z* dimension is integrated out; the circle shows the 15 Å radius of the dome. (c,d) Atomic-orbital representations of the same states. “S(*u*)” and “S(*l*)” stand for all atoms constituting the upper and lower S plane, respectively. (e) Valley contributions to the eigenstates close to the band edges in both UL and CL deformations. The color indicates the Γ component, i.e., the imaginary part of the complex valley polarization.

The VBM gap state around Γ, hosting two electrons, is highly localized at the apex [Figure 4a] and disproportionately constituted by p orbitals of the upper S plane [Figure 4c]. The asymmetry between upper- and lower-plane contributions is a manifestation of the curvature-induced loss of the basal σ_h_ reflection plane contained in the *D*_3*h*_ point group of flat 1L-MoS_2_, which enables mixing between the odd and even states.^45^ Hence, the high curvature at the top of the bubble causes the rehybridization of the states around the local maximum at Γ. It leads to a relocalization of the gap state toward the outer surface of the slab, giving rise to an electric dipole moment^46^ and increasing the material’s tendency to interact with adsorbates.^47^ The gap state formation occurs in both UL and CL structures, although the splitting is larger in the CL case, which features strain and curvature singularities. We note that the curvature and thus the orbital character and the separation between the gap and continuum states can be manipulated externally by applying global strain (Figure S5) or an electric field.^48^ The dispersive states below the localized state at Γ are strongly distorted with respect to the flat system with the maximum being displaced from the high-symmetry point. Tracking the evolution of the band structure upon increasing the curvature reveals that this distortion and the gap state split-off result from conflation of the zeroth-order band with the perturbative first-order replica band (Section S2). Such mixing implies a high probability of intravalley scattering for the corresponding holes (Section S3).

In the K valleys, too, satellites form around the zero-order bands, reflecting perturbative wave function components due to the deformation potential. The intersection between the main and secondary features occurs at the point  a distance π/(superlattice constant) away from K, which is also the zone center  of the quadratic mini BZ pertaining to the two-dimensional bubble array^49^ [inset of Figure 2b]. Contrary to the Γ holes, very little mixing takes place between their K counterparts, which would open an energy gap at the supercell zone edge.^50^ This effect can be observed in the conduction band, mainly with CL. Here, the lower portion of the band, including the CBm, is split off, forming an isolated band,^51^ which could give rise to unusual and highly doping-dependent charge carrier dynamics. In the case of isolated or randomly distributed defects, the gap opening is again an indication of substantial intravalley scattering. This potentially poses a limiting factor for low-temperature carrier mobility in n-type devices, especially with the electron-trapping nature of the deflections. The split-off miniband corresponds to semilocalized states [Figure 4b] with a strongly enhanced contribution of the  orbital of the central Mo atom [Figure 4d]. This effect is not observed for the smooth UL bubble, where the CBm remains in a delocalized Bloch state (Figure S8).

The deformation is thus very notable for holes at Γ and has a moderate (→UL) to strong (→CL) effect on electrons at K but barely influences holes at K. We conjecture that this is connected to the out-of-plane extension of the orbitals, which increases the susceptibility to curvature-induced rehybridization. Γ holes have a substantial S p character and are thus the most protruding; the CBm wave functions are mostly made up of Mo  orbitals, which also have a significant out-of-plane component; the holes at K correspond to Mo d_*xy*_ and  states and are oriented in plane.

#### Intervalley Scattering

Having disclosed processes and perturbations occurring within valleys, we now turn to the intervalley phenomena. Scattering between or mixing of different valleys involves large momenta of the order 2π/(lattice constant), which can be provided by atomic-scale variations of displacements and strain. We investigate our structures with regard to such phenomena by determining the contributions from different valleys to the eigenstates around the band edges. To this end, we compute the complex valley polarization of states close to the band edges according to eq 8, allowing us to distinguish contributions from the K, −K, and Γ valleys.

In the UL bubble (Figure 4e), most frontier states are neatly valley-polarized, i.e., can be unambiguously attributed to one of the three valleys. This implies a low probability of intervalley transitions, which are mediated by states with simultaneous contributions from multiple valleys. The absence of such processes can partly be ascribed to the lack of large-momentum components in the smooth UL deformation potential. However, valley polarization is largely maintained also in the valence band of the sharp CL structure, indicating the presence of another inhibiting mechanism. Indeed, the valley index of the holes in 1L-TDMCs is known to be protected by spin–valley coupling:^52^ the equal but opposite ∼150 meV splitting of the valence band at K and −K due to spin–orbit coupling leads to valence states at the same energy having antiparallel spins within the K and −K valleys. An elastic scattering event must, therefore, flip the spin. While this is not impossible in curved 1L-TMDCs, the polarization of the states indicates that such processes are rare even in the presence of sharp perturbations. Transitions are more commonly deeper in the valence band, where no spin flip is required. Some states have components from all three valleys, including opposite spins in the K and −K valleys. These are possibly related to hypothesized phonon-mediated K ↔Γ ↔ −K cascade scattering in flat 1L-MoS_2_.^53,54^

In the conduction band, spin–valley locking is less effective as the spin–orbit split at the CBm amounts to only a few meV, opening up the possibility for spin-preserving intervalley transitions close to the band edge [Figure 4e]. However, the UL bubble is too smooth to provide sufficient momentum to couple states across the BZ, leaving the CBm almost completely polarized. In the CL case, on the other hand, the conduction states are completely mixed between the K and −K valleys, which goes hand in hand with the above-mentioned increased real-space localization of the CBm.

Valley mixing and spatial confinement have several important implications. The former means that the defects cause intervalley scattering, similar to short-wavelength phonons at higher temperatures.^32,55^ The diminishing effect on the K/–K valley polarization of electrons and excitons renders them problematic in the relatively young field of valleytronics, which aims at instrumentalizing this degree of freedom.^56−58^ On the positive side, valley symmetry breaking has also been conjectured to be the enabling factor for single-photon emission in tungsten diselenide,^20^ in which valley-conserving photoluminescence is spin-forbidden.^59^

Continuing along these lines, we make some additional considerations regarding the optoelectronic properties of the deformed systems based on the electronic structure. The first pertains to the polarization of emitted light, which is likely influenced by valley mixing. Hexagonal two-dimensional semiconductors without inversion symmetry are subject to the selection rule that transitions at K/–K only couple to in-plane circularly polarized σ^+^/σ^–^ light.^60^ With a CBm equally mixed between the K and −K states, it stands to reason that emitted light has a mixture of σ^+^ and σ^–^ polarization, resulting in increased linear polarization.^61^ This has been observed in numerous luminescence measurements.^1,3,24,61,62^ Due to the 3-fold rotational symmetry of the deformation, the transition dipole moment does not have a well-defined orientation within the plane, which would require additional symmetry-breaking perturbations. Corresponding observations have been made in less symmetric deformations, where the polarization direction tends to align with structural features.^24^

The second point concerns the expected character of excitons. As the atomic orbitals constituting the K-band edges are the same in flat and deformed structures, the defect excitons are expected to share many similarities with the A exciton of the flat material.^63^ However, they are anticipated to have different sizes, with trapped excitons being smaller. This is the result of the more localized wave functions, especially around the CBm [Figure S8c].

The local band gap reduction translates almost entirely into the optical gap, according to ab initio calculations based on many-body perturbation theory.^44^ The gap reduction found in this work (70 meV for CL) is consistent with the luminescence energy difference of single-photon emission with respect to valley exciton recombination found, e.g., in refs (1)–^5^ (50–60 meV). This agreement, together with the broken valley symmetry, the (partial) linear polarization of the emission, and the increased exciton localization, suggests that sharp deformations are indeed sources of single-photon emission. Since it is conceivable that these defects arise accidentally due to substrate roughness and trapped particles, it is possible that they are relevant even in scenarios where no strains are deliberately introduced. In the particular case of 1L-MoS_2_, the emission strength is rather low since the VBM is at Γ, and the bright lowest-energy K exciton has the same energy as its dark counterpart,^59^ implying competitive radiative recombination of valley excitons. However, as we expound below, the impact of the perturbation on the electronic structure is largely symmetry-determined and thus manifests similarly in other 1L-TMDCs that do not exhibit these two compromising features.

#### Interactions with Sulfur Vacancies

To complete our discussion, we extend our analysis to include single S vacancies (SVs), which are commonly encountered point defects in 1L-MoS_2_^64,65^ but can also be manufactured by irradiation with high-energy particles.^66−68^ To appreciate their effect on the electronic structure, we first circle back to the band structure of flat, relaxed (0%) 1L-MoS_2_ [Figure 2b]. In agreement with previous reports,^69,70^ we find SVs to give rise to a shallow occupied and a deep, degenerate unoccupied gap state. These states transform according to the *A*_1_ and *E* representations of the *C*_3*v*_ point group, respectively, which characterizes the local crystal field symmetry. Upon application of global tensile strain (2%), the *A_1_* state moves into the continuum, reinstating the delocalized nature of the VBM, with no significant interactions occurring (Figure S9).

**Figure 5:**
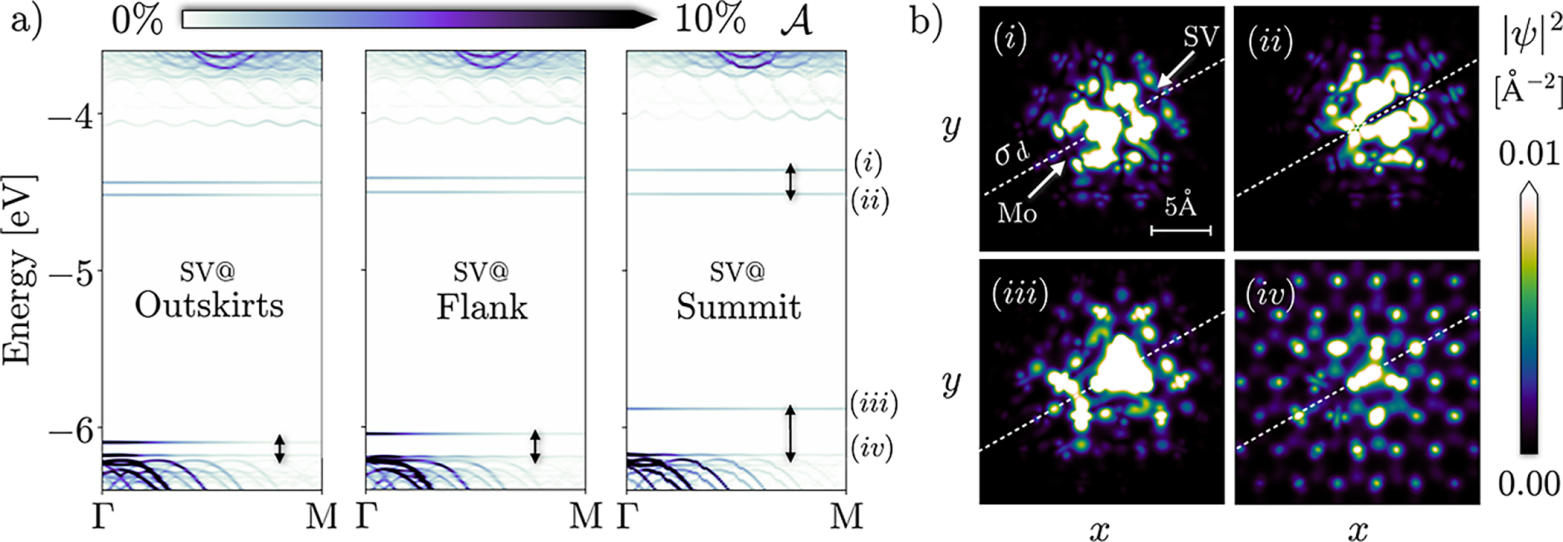
(a) Spectral function  for CL deformations with sulfur vacancies (SVs) in three different sites [cf. Figure 1b for their exact positions]. (b) Real-space representations of the four gap states in the bubble with the vacancy on the summit, labeled in the rightmost panel in (a), with the *z* dimension integrated out. The reflection plane σ_d_ is indicated by a dashed line, while the positions of the central Mo atom and the sulfur vacancy are highlighted by arrows.

For the study of SVs close to deflections, we consider a CL structure with three different defect sites [Figure 1b]. In each case, the *E* state is split into two [Figure 5a], separated by a gap that is largest when the SV is at the summit, i.e., directly adjacent to the central Mo atom. The split reflects the reduction of the local crystal field symmetry from *C*_3*v*_ to *C*_*s*_, a point group that features only a reflection plane σ_d_ perpendicular to the basal plane and running through both the central Mo atom and the SV. The *E* representation can be disassembled into *C*_*s*_ irreducible ones as *E* = *A*′ + *A*″. Thus, one of the new states is symmetric with respect to σ_d_ reflection [*A*′, Figure 5b(i)], and the other antisymmetric, as manifested in the nodal plane [*A*″, Figure 5b(ii)], with the latter having lower energy.

The occupied SV-related *A*_1_ state mixes with the curvature-induced gap state. On the one hand, this is unsurprising since the latter has been established to have prevalent S p character and thus can be expected to be strongly affected by a corresponding vacancy. On the other hand, it is a stark contrast to the situation in the strained flat system, where the *A* state does not interact with other states to a significant degree. The mixing is strongest when the point defect is near the apex, in which case the interaction-induced split amounts to several hundred meV. Inspecting the real-space representations, it is found that the upper one is strongly localized [Figure 5b(iii)], while the lower one has a relatively large spatial extension [Figure 5b(iv)]. The interaction pushes the state very close to the lower bands at Γ, where it assumes some character of the continuum states and becomes semiextended. Both occupied gap states have *A*′ symmetry. Relating the higher-energy one to the two unoccupied states, we predict the existence of two cross-linearly polarized optical transitions, with the lower-energy one (involving the *A*″ state) having a transition dipole with azimuthal orientation.

#### Extension to Other TMDCs and Multilayers

Before concluding, we address two points regarding the generality of our results. While we have focused on 1L-MoS_2_ in this work, we expect the same qualitative results in other 1L-TMDCs, since the crystal structures and the band characters are comparable.^47^ To confirm this, we check explicitly one other representative, namely, molybdenum diselenide [Figure S10a], indeed finding a remarkably similar picture. The main difference consists of the gap state no longer constituting the VBM as the Γ valley lies significantly lower in nonsulfuric 1L-TMDCs.^47^ A final potential point of interest is the modeling of the TMDC as a single layer. This is appropriate when the material is suspended or adsorbed on an incommensurate substrate. However, if bubbles form at the surface of a bulk TMDC crystal or when multiple sheets are stacked on a substrate, wave functions of different layers may hybridize. These interactions significantly elevate the Γ valley to the point of becoming the VBM and turning the band gap indirect.^71^ In the context of the present results, this raises questions about the curvature-induced gap state, which is also located at Γ. Comparing band structures of monolayer and bilayer structures suggests that interlayer interactions weaken the rehybridization effect, which no longer entails complete localization of a separated gap state [Figure S10b]. The same effect is expected to occur in heterojunctions, i.e., bilayer systems composed of two different 1L-TMDCs, which can similarly hybridize at Γ.^72^ However, it is unclear to which extent this affects interlayer phenomena, e.g., interlayer exciton formation, since these are usually determined by the K valleys, where interlayer interactions are significantly weaker.^72^

## Summary and Conclusions

In summary, we investigated with density functional theory the electronic structure of both smooth and sharp atomic-scale deformations in monolayer molybdenum disulfide, generating quantum-dot-like structures. For both types of defects, the associated curvature gives rise to an occupied gap state with strong in-plane and outer-surface localization. This feature is intimately related to the high probability of intravalley scattering. If the deformation is sharp, the wave function associated with the conduction band minimum at K becomes increasingly centered on the exposed Mo atom, furnishing it with enhanced single-atomic  character. In reciprocal space, the localization is reflected in K/–K valley mixing and, upon periodic spatial arrangement, the formation of energetically separated subbands. We conjecture that many observations of single-photon emission in different transition metal dichalcogenides can be traced back to such defect states. The valley mixing also implies the possibility of intervalley defect scattering of electrons, while the valley polarization of holes remains protected by spin–valley coupling. Finally, we discovered significant interactions between curvature- and vacancy-induced states, which can potentially be leveraged for further tailoring of the local electronic structure. We hope that this work provides inspiration for new approaches to creating artificial atoms in transition metal dichalcogenides and contributes to the rationalization of already observed phenomena.

## Data Availability

Input and output files for constrained geometry optimizations of central- and uniform-load deformations are openly available free of charge at https://zenodo.org/records/10495941.

## Supporting Information Available

Role of the chosen exchange–correlation functional, localized bands, periodic boundary conditions, and real-space localization of the Bloch states (PDF)
