# Supplementary material for: Quantum Dots in Transition Metal Dichalcogenides Induced by Atomic-Scale Deformations
Source: ACS Photonics. 2024 Jan 16;11(2):586–95. doi: 10.1021/acsphotonics.3c01470 (PMC10885200; doi:10.1021/acsphotonics.3c01470)
Supplement: Supplementary file 1 — ph3c01470_si_001.pdf [file ph3c01470_si_001.pdf]

SUPPORTING INFORMATION

Quantum Dots in Transition Metal Dichalcogenides Induced by Atomic-Scale Deformations

Jannis Krumland,[*,†,‡] Stefan Velja,[†] and Caterina Cocchi[*,†,‡]

†*Institute of Physics, Carl von Ossietzky Universität Oldenburg, 26129 Oldenburg, Germany*

‡*Physics Department and IRIS Adlershof, Humboldt-Universität zu Berlin, 12489 Berlin, Germany*

E-mail: jannis.krumland@physik.hu-berlin.de; caterina.cocchi@uni-oldenburg.de

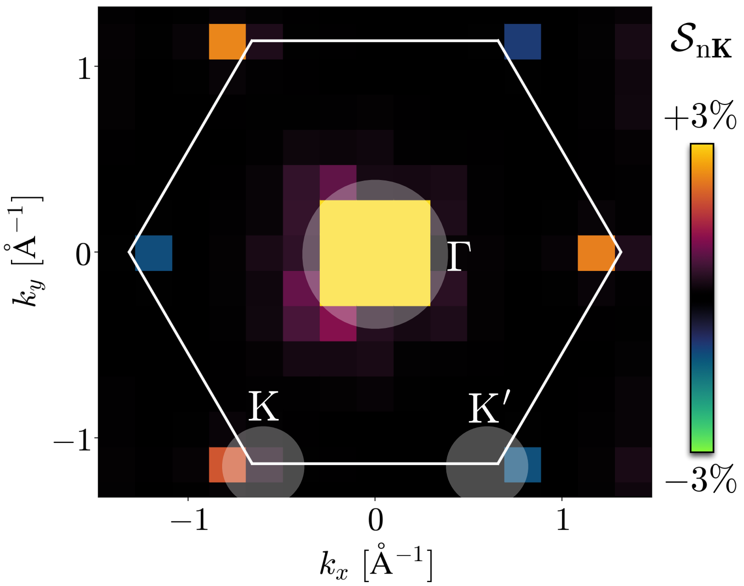

Figure S1: Spin-resolved spectral function $\mathcal{S}_{n\mathbf{K}}$ for state $n = 3108$ of the central-load structure, which lies 200 meV below the gap state ($n = 3120$). Every tile represents a **k**-point mapping onto the supercell wavevector $\mathbf{K} = 0.01\pi(1/|\mathbf{A}_1|, 1/|\mathbf{A}_2|)$. The small displacement of $\mathbf{K}$ with respect to supercell $\tilde{\Gamma}$ is applied to break zone-center-specific symmetries. The feint circles indicate the integration domains used to compute the populations $P_{n\mathbf{K}}(\mathrm{K})$, $P_{n\mathbf{K}}(-\mathrm{K})$ and $P_{n\mathbf{K}}(\Gamma)$, which enable the determination of the valley polarization $\mathrm{VP}_{n\mathbf{K}}$: all tiles touched by the circles are included, amounting to 9 tiles each for K and −K, 21 for Γ. Note that the absolute-valued $\mathcal{A}_{n\mathbf{K}}$ are considered for this procedure, not the spin-polarized version $\mathcal{S}_{n\mathbf{K}}$ on display here.

S1 Effect of Hybrid Functional

To check whether hybrid functionals give rise to differences in the electronic structure beyond a rigid gap opening in inhomogeneously strained 1L-TMDCs, we calculate and compare the band structure of a 1L-MoS_2 wrinkle with PBE and HSE[1,2] [Fig. S2b)-d)]. It is found that HSE opens the gap by an additional amount at the M point, which influences the slope of the bands between the high-symmetry points. Thus, the higher-level result brings about more than a mere rigid shift. However, the difference is only quantitative, not qualitative, and the M point is irrelevant in our discussions. Thus, we employ the computationally more efficient PBE.

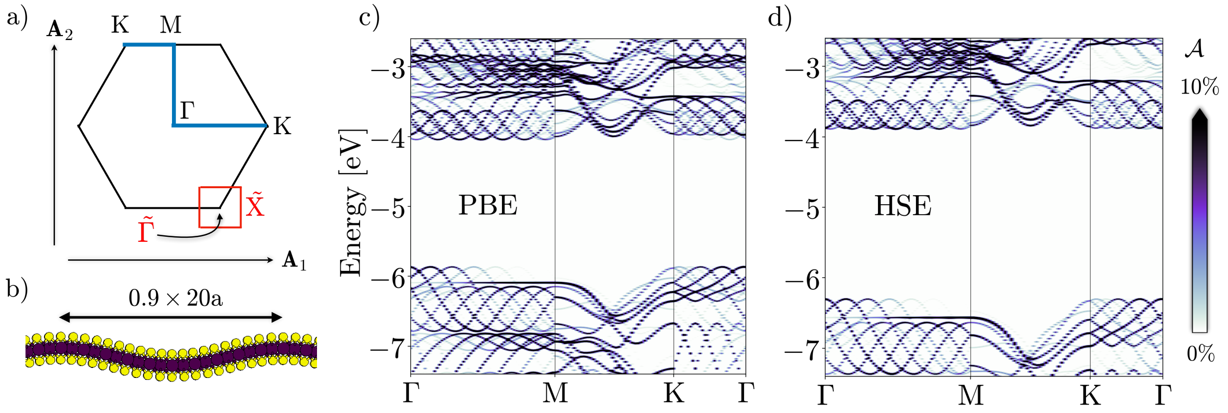

Figure S2: a) Primitive-cell Brillouin zone (delimited by black lines) and path along which the band structure for the bubbles is calculated. The small zone outlined in red pertains to the superlattice, which is spanned by the real-space vectors $\mathbf{A}_1$ and $\mathbf{A}_2$. b) Side view of a 10% compressively strained MoS_2 wrinkle in $20 \times \sqrt{3}$ cell, which is used as a test system to compare PBE (c) and HSE (d) results.

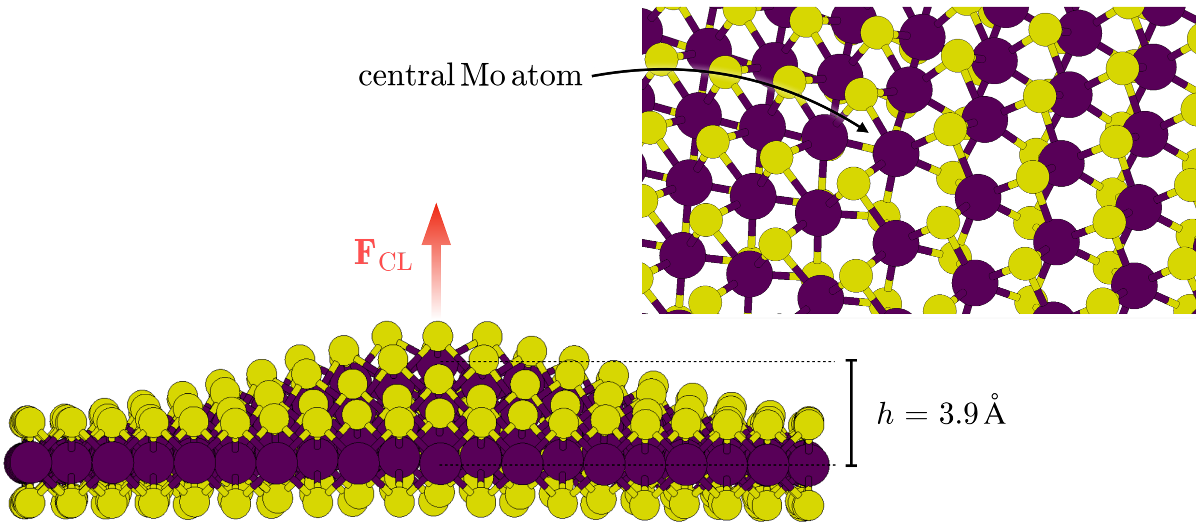

Figure S3: Optimized geometry of a deformation arising as a result of strong forces being applied to the three S atoms below the central Mo atom, which is elevated to a height $h = 3.9\,\mathrm{\AA}$ above the base plane. The zoom into the central region shows intact Mo-S bonds and no reconstruction.

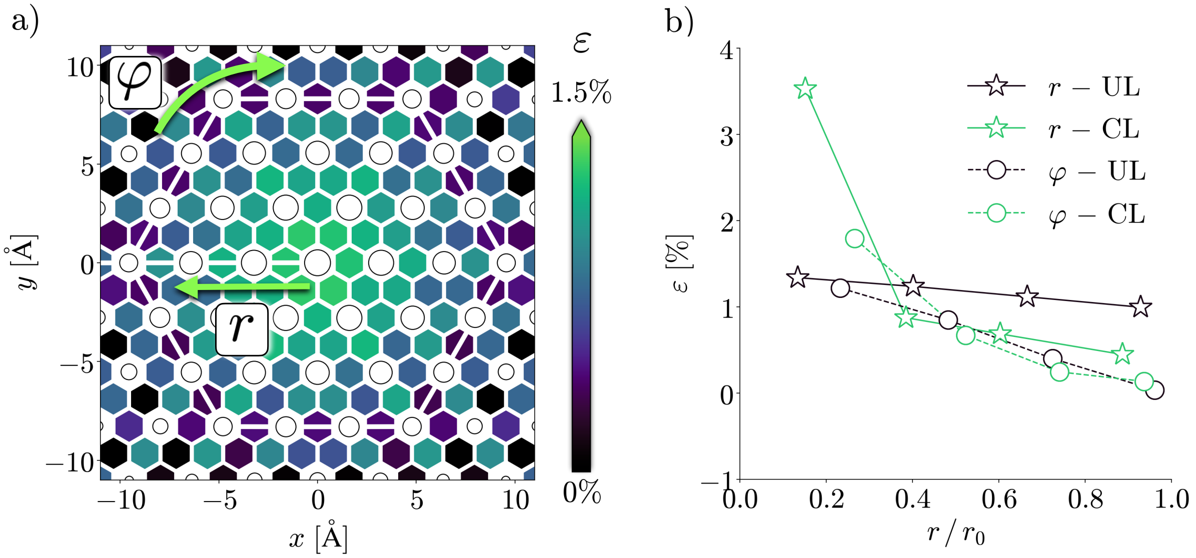

Figure S4: a) Strain distribution in the uniform-load bubble. For each pair of Mo atoms, the strain is calculated as $\varepsilon = (d - d_0)/d_0$, where d is the distance between them and d_0 is the lattice constant. A radial (r) and a circumferential path (φ) are drawn. b) Strain along r and φ directions for uniform (UL) and central load (CL) bubbles. For each value r/r_0, where r_0 is the radius of the bubble, the r strain is calculated by averaging the six values obtained by a C_6 rotation of the path segments shown in panel a); the φ strain is calculating by averaging over all values in the hexagonal φ path drawn in panel a).

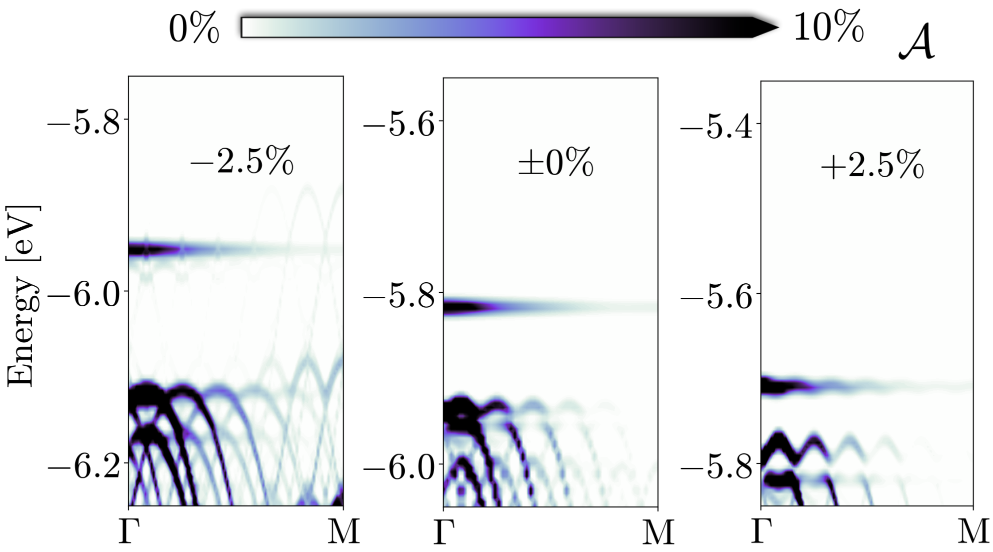

Figure S5: Local valence band maximum at Γ in a central-load bubble with compressive (-2.5%) and tensile (+2.5%) global strain applied, compared to the unstrained case (±0%). The same Mo are fixed at the same heights in all cases.

S2 Side Bands and Localized States

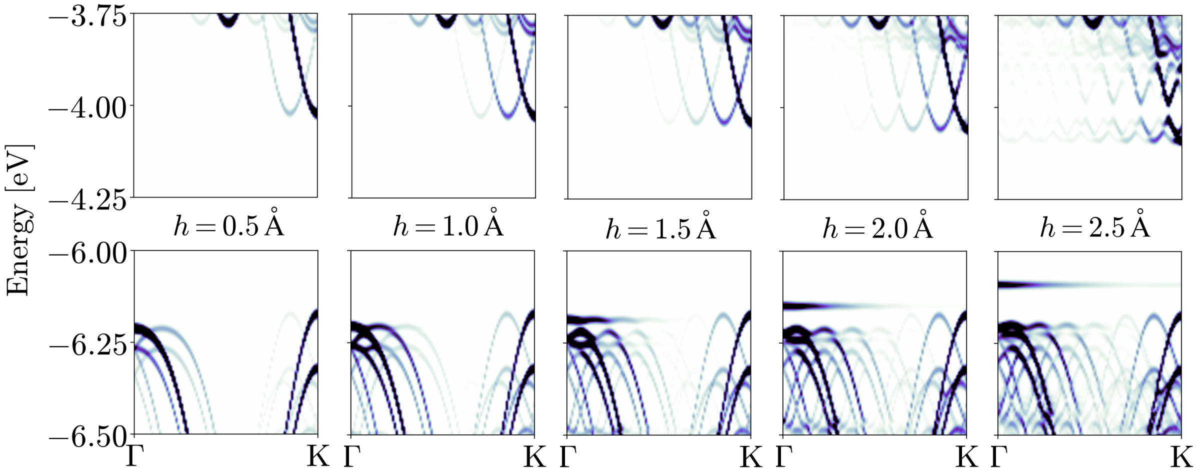

Figure S6: Evolution of the band edges in a central-load protrusion upon increasing the height h of the central Mo atom.

Comparing the band structure of pristine 1L-MoS_2 [Fig. 2b)] with the unfolded ones of the bubbles (Fig. 3), the most salient differences are the emergence of satellite bands and band splittings in the latter. As it turns out, these two are closely related. To see this, it is instructive to investigate CL bubbles with increasing heights $z = h$ of the central Mo above the base plane ($z = 0$) (Fig. S6).

When $h = 0.5$ Å, the perturbation is rather weak and the main band prevails [panel a)]. Some first-order side bands can clearly be spotted, but they appear superposed without interactions. These side bands are not entirely new states, but represent perturbative components obtained by the main wave functions around Γ that have the same energy. The main band is separated from its associated side bands by multiples of $2\pi/l$, where l is the distance between bubble replica in neighboring supercells, for reasons laid out in the next section. Wave functions not having a well-defined wave vector is a manifestation of the loss of unit-cell translational symmetry in the deformed structure. Once the perturbative components become substantial, the supercell wave functions can turn into wave packets. Thus, electrons and holes are no longer necessarily delocalized as in the flat material, but may prefer certain subregions of the supercell, *e.g.*, the top of the dome. For $h = 0.5$ Å, the

side bands are still feeble and cannot influence the main bands to a significant degree. This changes once h is increased to 1 Å [panel b)]. The intensity of the first side band increases, and even a faint second-order satellite emerges at twice the $\mathbf{k}$-distance. At this point, interactions start to take place at the intersection between the main band and the first side band, resulting in a mixing of the $|\mathbf{k}| = \pm\pi/l$ states. Further elevating the central Mo to $h = 1.5$ Å [panel c)], the interactions are so strong that they cause a band split, giving rise to standing waves localized in different parts of the supercell. Standing waves have forward- and backward-propagating components, implying a large intravalley scattering cross section for corresponding charge carriers. Finally, reaching heights of $h = 2 - 2.5$ Å [panels d)-e)], the upper state straightens out to become completely dispersionless, turning it into the gap state strongly localized at the top of the dome. While spread significantly in reciprocal space, it hosts just two electrons with opposite spin. The round probability distribution in real space is reflected in a similarly shaped $\mathbf{k}$-distribution around Γ.

S3 Isolated Entities *versus* Arrays

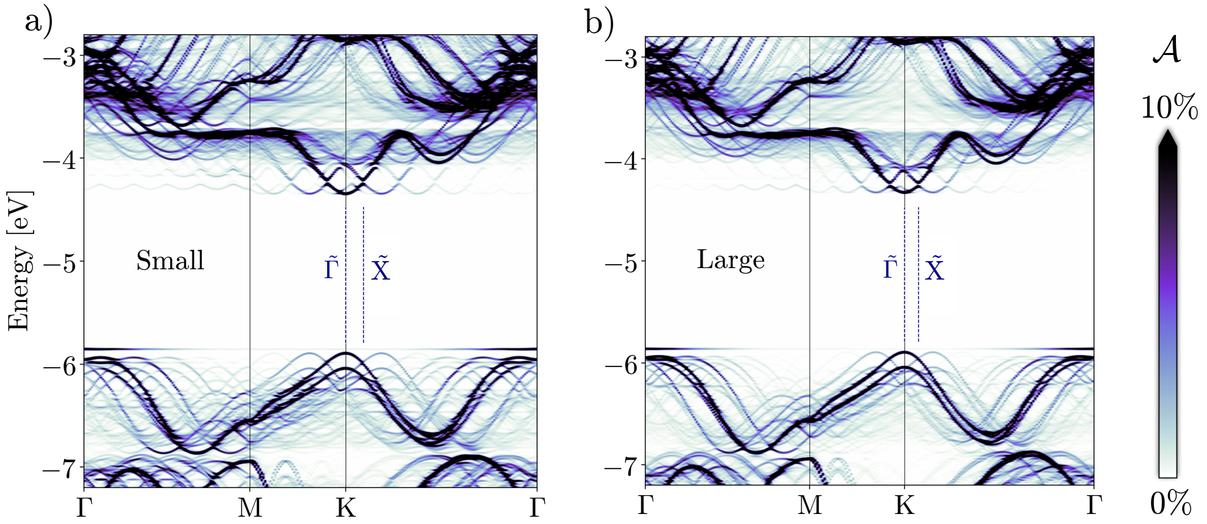

Figure S7: Unfolded band structure for the same $r = 12$ Å, $h = 1.85$ Å central-load structure in $8 \times 5\sqrt{3}$ (a) and $10 \times 6\sqrt{3}$ (b) supercells.

In our simulations, we enforce periodic boundary conditions. Thus, we effectively simulate not an isolated bubble, but an two-dimensional superlattice of them, which is spanned by the supercell lattice vectors $\mathbf{A}_1$ and $\mathbf{A}_2$. In the following, we assume $|\mathbf{A}_1| = |\mathbf{A}_2|$, although this is not strictly true, and refer to these lenghts as l. As pointed out in the previous section, the perturbative side bands in the unfolded band structures are displaced by $n \times 2\pi/l$ with respect to the main bands, where n is an integer. This can be verified by inserting the same mCL bubbles into two different supercells (Fig. S7), thus varying l but leaving all other parameters unchanged. This gives rise to tangible differences in the electronic properties, *e.g.*, in the energy gap between the CBm and the first subband above it. Consequently, such properties have to be regarded as features of the superlattice rather than of isolated bubbles. We consider the interactions taking place at π/l away from the CBm, which lead to a small split of the lowest conduction band into an upper and a lower branch. These correspond to standing waves localized in complementary parts of the supercell. The dependence on the spacing l can be explained through the superlattice's Bragg condition, which is met for electrons with different $\mathbf{k}$ due to the difference in l. However, it is clear that a periodic array of centers can only effectively reflect waves if these centers have a large scattering cross section. Consequently, the splits are indicators of intravalley scattering, since the interactions occur between different states within the same valley.

S4 Real-Space Localization

We define an indicator for the real-space localization (RSL) of Bloch states as

$$\mathrm{RSL}_{n\mathbf{K}} = 2\left[\sum_{\sigma}\int_{|\mathbf{r}_\parallel|<r_{1/2}} \mathrm{d}^2 r_\parallel \int \mathrm{d}z\, |\psi_{n\mathbf{K}}(\mathbf{r},\sigma)|^2 - \frac{1}{2}\right]. \tag{S1}$$

Here, $r_{1/2}$ is the radius of a circle whose area is half that of the simulation cell,

$$r_{1/2} = \left(\frac{|\mathbf{A}_1||\mathbf{A}_2|}{2\pi}\right)^{1/2}, \tag{S2}$$

and which is centered at the central Mo atom ($x = y = 0$). $\mathrm{RSL}_{n\mathbf{K}}$ assumes values between -1 and 1, where a value of 0 indicates Bloch-like character, *i.e.*, a wave function with uniform density throughout the supercell. For positive $\mathrm{RSL}_{n\mathbf{K}}$, the corresponding electron prefers the central, deformed region; for negative ones, the it is more likely to be found in the flat periphery. Here we consider the zone center of the supercell BZ, *i.e.*, $\mathbf{K} = \tilde{\Gamma}$.

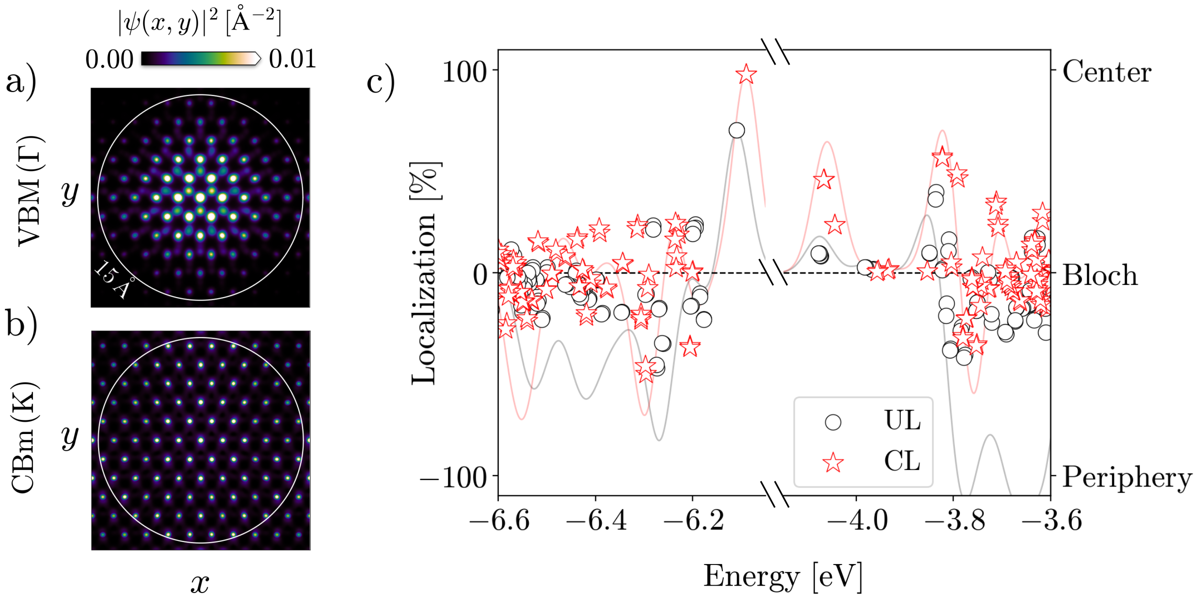

Figure S8: a)-b) Real-space representations of the valence band maximum and the conduction band minimum in the uniform-load structure. c) Real-space localization of the deformation eigenstates at $\tilde{\Gamma}$ around the band edges, calculated according to Eq. (S1).

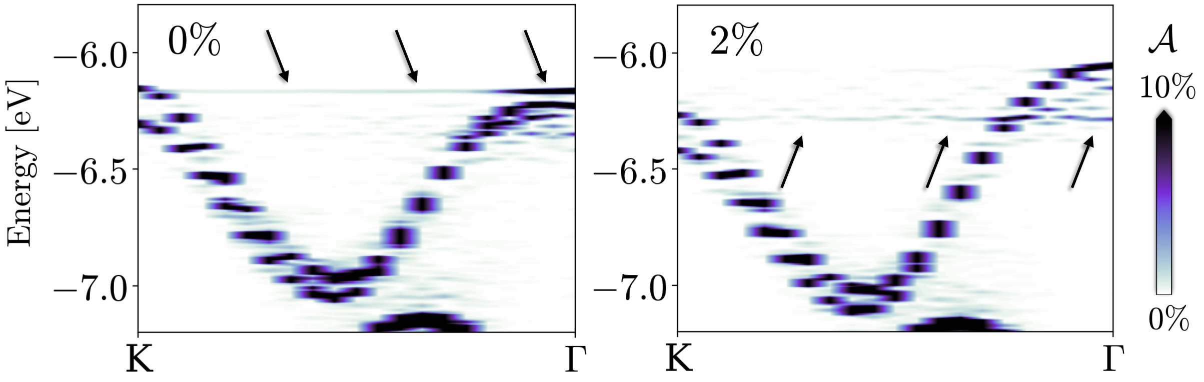

Figure S9: Unfolded band structure near the valence band maximum in flat 1L-MoS_2 without (0%) and with tensile strain (2%) with a sulphur vacancy. The arrows highlight the occupied A_1 state.

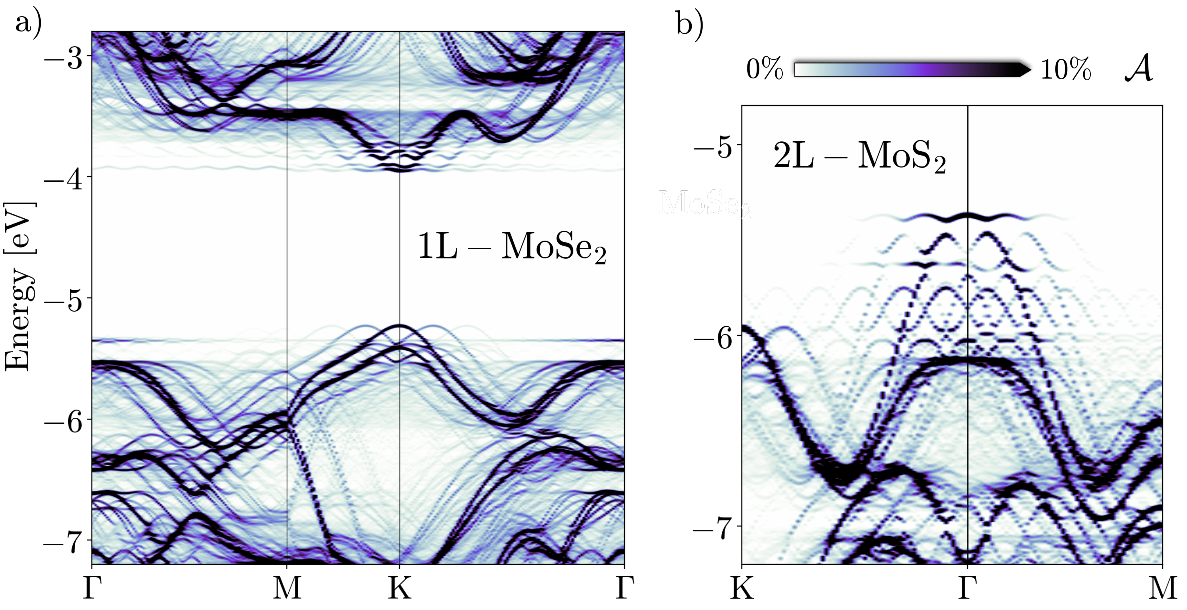

Figure S10: Valence band maximum at Γ for bilayer MoS_2, where a $r = 12\,\text{Å}, h = 1.85\,\text{Å}$ central-load structure is situated in the upper layer. Spin-orbit coupling is not taken into account. The system is contained in a $8 \times 5\sqrt{3}$ supercell.

References

(1) Heyd, J.; Scuseria, G. E.; Ernzerhof, M. Hybrid functionals based on a screened Coulomb potential. *J. Chem. Phys.* **2003**, *118*, 8207–8215.

(2) Heyd, J.; Scuseria, G. E.; Ernzerhof, M. Erratum: "Hybrid functionals based on a screened Coulomb potential" [J. Chem. Phys. 118, 8207 (2003)]. *J. Chem. Phys.* **2006**, *124*, 219906.
